# Pseudosarcomatous Fibromyxoid Tumor of the Prostate

**DOI:** 10.1100/tsw.2011.87

**Published:** 2011-05-05

**Authors:** Gokhan Atis, Cenk Gurbuz, Murat Can Kiremit, Bayram Guner, Ebru Zemheri, Turhan Caskurlu

**Affiliations:** ^1^ 2nd Urology Clinic, Göztepe Training and Research Hospital, Istanbul, Turkey; ^2^ Pathology Clinic, Göztepe Training and Research Hospital, Istanbul, Turkey

**Keywords:** fibromyxoid, prostate, pseudosarcomatous, inflammatory pseudotumor

## Abstract

We present the case of a 61-year-old patient who was evaluated for benign infravesical obstruction due to a pseudosarcomatous fibromyxoid tumor of the prostate. This entity is rare and difficult to distinguish from a malignant lesion. A discussion of the pathological features and a review of the literature are given.

## INTRODUCTION

Pseudosarcomatous fibromyxoid tumor of the prostate is a rare benign lesion that has previously been mistaken for a malignant prostatic sarcoma. It is important to distuinguish this lesion from a malignant lesion in order to avoid unnecessary radical procedures. We present a case in which the patient was treated with transurethral resection of the prostate. Although these tumors often recur, the patient remained asymptomatic without the need for additional treatment.

## CASE REPORT

A 61-year-old male patient was admitted to our clinic with acute urinary retention, requiring bladder catheterization and subsequent negative catheter removal tests. The prostate-specific antigen level was 1.73 ng/ml. Digital rectal examination showed an enlarged prostate without nodules that was filling 50% of the rectum. He had a smoking history of 40-years duration. He had no history of pelvic irradiation, instrumentation, or previous malignancy. No hydronephrosis, renal or bladder lesions were noted at urinary ultrasonography. The patient underwent transurethral resection of the prostate (TURP). Postoperatively, the patient voided normally with no evidence of recurrence on follow-up of over 1 year. Pathology revealed pseudosarcomatous fibromyxoid tumor with no evidence of malignancy.

The TURP specimen measured about 20 g in weight and had gross nodular characteristic. Microscopically, the tumor was composed of spindle cells arranged in a myxoid background with numerous inflammatory cells (Figs. 1 and 2). Rare mitotic figures with no significant atypia were noted. Immunohistochemical staining was positive for vimentin (Fig. 3). Staining was negative for cytokeratin and

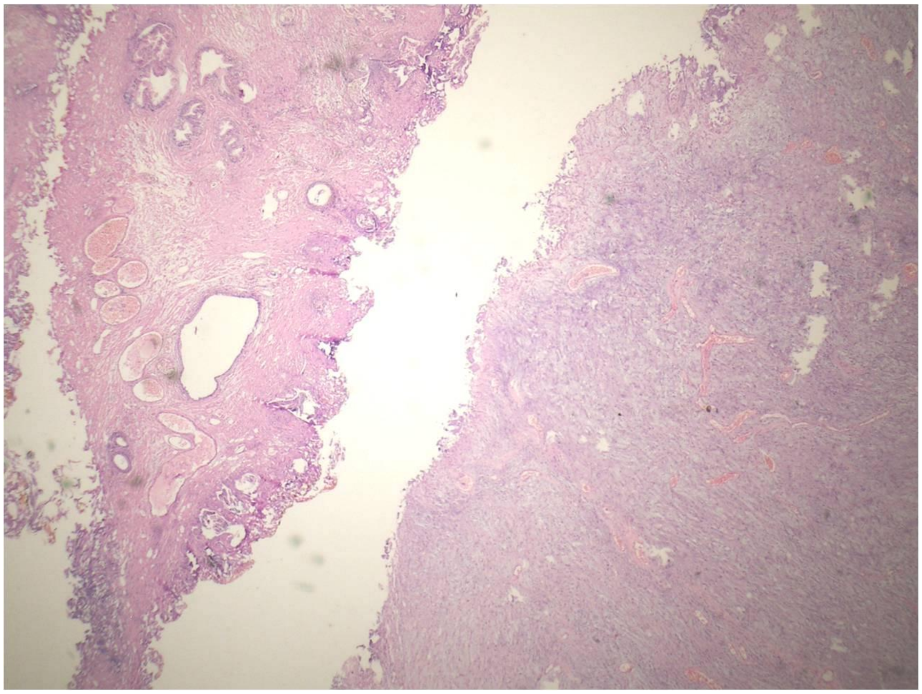

**FIGURE 1.** Overview of the dominating structures (hematoxylin-eosin staining; original magnification ×100).

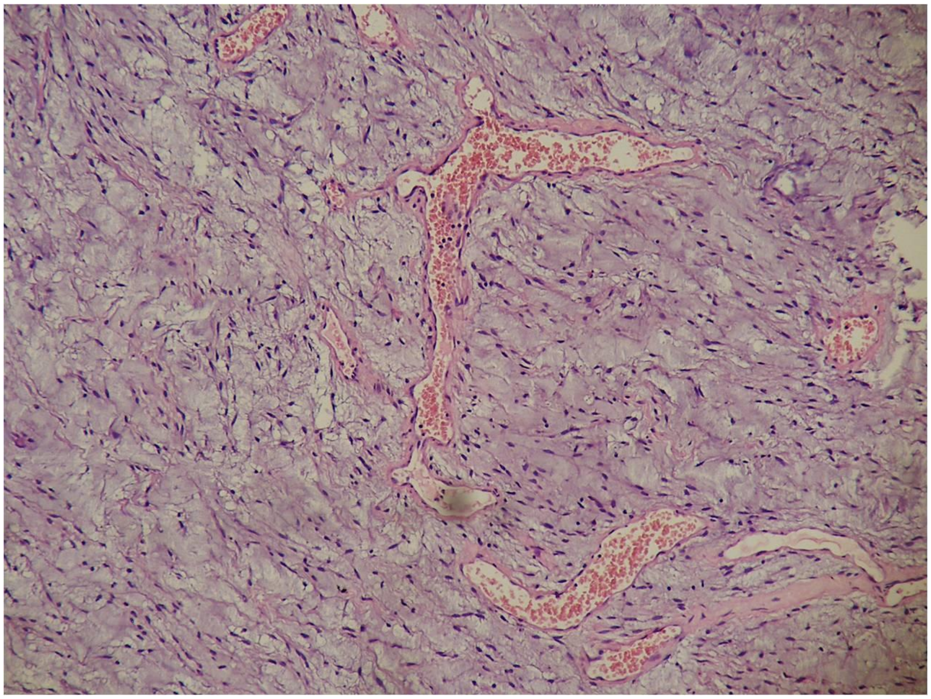

**FIGURE 2.** An image of spindle cells in a myxoid stroma (hematoxylin-eosin staining; original magnification ×100).

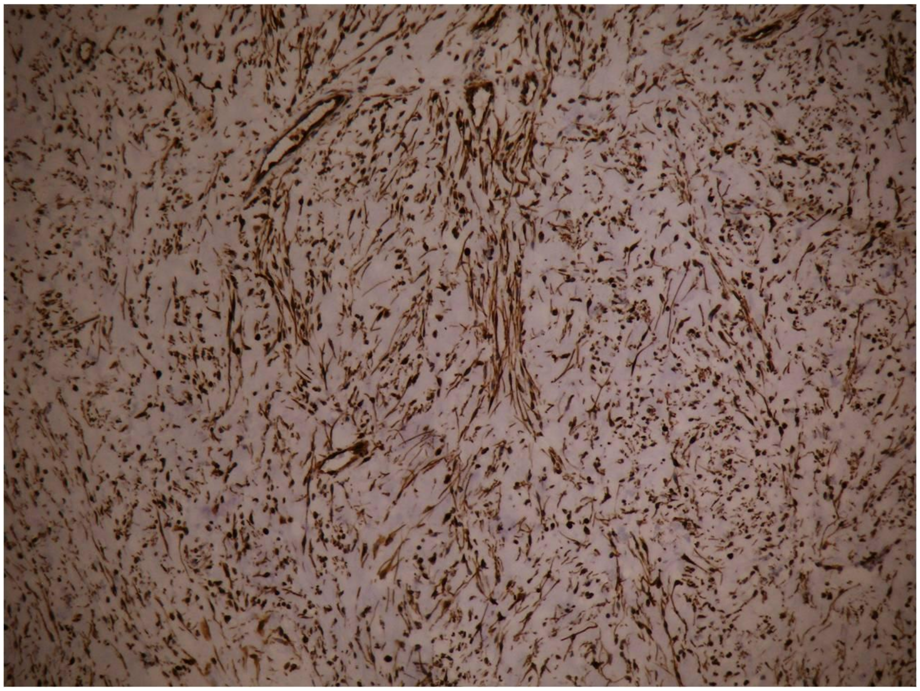

**FIGURE 3.** Staining for vimentin (original magnification ×100).

CD 34. The proliferation activity was low as assessed using the proliferation marker Ki-67. Ki-67 level was lower than 1%.

## DISCUSSION

Pseudosarcomatous fibromyxoid tumors are rare lesions with uncertain pathogenesis. These tumors can also be termed inflammatory pseudotumors, inflammatory myofibroblastic tumors, pseudosarcomatous myofibroblastic tumors, fibromyxoid pseudotumors, pseudomalignant spindle cell proliferation, and nodular fasciitis[1].

In 1980, Roth first described a spindle cell lesion located in the urinary bladder using the term "reactive pseudosarcomatous response"[2]. In 1984, Hafiz et al. described a similar lesion of the prostate[3]. Ro et al. reported this type of lesion in the urinary bladder and prostate, and was the first to use the term "pseudosarcomatous fibromyxoid tumor"[4,5]. Inflammatory pseudotumors have also been observed in various other organs throughout the body, such as the ureter[6], vagina[7], and urethra[8]. Tsuzuki et al. described an expression of anaplastic lymphoma kinase (Alk)-1 in these tumors[9]. The Alk gene was originally identified in anaplastic large-cell lymphoma carrying the t (2;5) (p23;q35) translocation[10].

The etiopathogenesis of pseudosarcomatous fibromyxoid tumors is unknown. In most of the reported cases, common associations were smoking, previous instrumentation, and surgery. In our patient, smoking was the only risk factor for pseudosarcomatous tumor.

The differential diagnosis should include sarcomatoid urothelial carcinoma, leiomyosarcoma, and rhabdomyosarcoma because of the mesenchymal proliferation[11]. It is well known that sarcomas show marked cytologic atypia, atypical mitotic figures, and nonmyxoid areas with marked increased cellularity in the spindle cell areas. These findings usually allow for a diagnosis of sarcomatoid carcinoma. Only a few cases of pseudosarcomatous tumors have been reported to be mitotically active[12]. Positive cytokeratin reactivity is also considered as a definitive feature of sarcomatoid carcinoma; however, most pseudosarcomatous fibromyxoid tumors could show pan-cytokeratin reactivity[13]. In our case, staining was negative for cytokeratin.

Postoperative spindle cell nodule, first described by Proppe et al., is another lesion that has a similar entity to a pseudosarcomatous fibromyxoid tumor[14]. This lesion originates from recent occurrences, such as invasive trauma, mainly cystoscopies or TURPs. In our patient, there was no history of instrumentation and pseudosarcomatous fibromyxoid tumor was diagnosed from the first resection.

Jensen et al. reported a case of pseudosarcomatous fibromyxoid tumor of the prostate. In their case, the patient underwent another TURP because of increasing lower urinary tract symptoms[15]. Similarly, Harik et al. recently studied 42 cases of pseudosarcomatous fibromyxoid tumor of the bladder and reported that some patients developed recurrences, but none had metastases[1]. One of the characteristics of pseudosarcomatous fibromyxoid tumor is its very rapid growth. In our case, the patient remained asymptomatic without the need for additional treatment.

In conclusion, pseudosarcomatous fibromyxoid tumors of the prostate are rare lesions, and it is important for the urologist and pathologist to distuinguish this benign process from a malignant lesion in order to avoid unnecessary radical procedures.

## References

1. Harik, L.R., Merino, C., Coindre, J.M., et al. (2006) Pseudosarcomatous myofibroblastic proliferations of the bladder. A clinicopathologic study of 42 cases. *Am. J. Surg. Pathol.* **30,** 787–794.
2. Roth, J.A. (1980) Reactive pseudosarcomatous response in urinary bladder. *Urology* **16,** 635–637.
3. Hafiz, M.A., Toker, C., and Sutula, M. (1984) An atypical fibromyxoid tumor of the prostate. *Cancer* **54,** 2500–2504.
4. Ro, J.Y., Ayala, A.G., Ordonez, N.G., et al. (1986) Pseudosarcomatous fibromyxoid tumor of the urinary bladder. *Am. J. Clin. Pathol*. **86,** 583–590.
5. Ro, J.Y., el-Naggar, A.K., Amin, M.B., et al. (1993) Pseudosarcomatous fibromyxoid tumor of the urinary bladder and prostate: immuno-histochemical, ultrastructural, and DNA flow cytometric analyses of nine cases. *Hum. Pathol*. **24,** 1203–1210.
6. Horn, L.C., Reuter, S., and Biesold, M. (1997) Inflammatory pseudotumor of the ureter and the urinary bladder. *Pathol. Res. Pract*. **193,** 607–612.
7. Guillou, L. and Costa, J. (1989) Postoperative pseudosarcomas of the genitourinary tract. A diagnostic trap. Presentation of 4 cases of which 2 were studied immunohistochemically and review of the literature. *Ann. Pathol*. **9,** 340–345.
8. Young, R.H. and Scully, R.E. (1987) Pseudosarcomatous lesions of the urinary bladder, prostate gland, and urethra. A report of three cases and review of the literature. *Arch. Pathol. Lab. Med.* **111,** 354–358.
9. Tsuzuki, T., Magi-Galluzzi, C., and Epstein, J.I. (2004) ALK-1 expression in inflammatory myofibroblastic tumor of the urinary bladder. *Am. J. Surg. Pathol*. **28,** 1609–1614.
10. Morris, S.W., Kirstein, M.N., Valentine, M.B., et al. (1994) Fusion of a kinase gene, ALK, to a nucleolar protein gene, NPM, in non-Hodgkin’s lymphoma. *Science* **263,** 1281–1284.
11. Montgomery, E.A., Shuster, D.D., Burkart, A.L., et al. (2006) Inflammatory myofibroblastic tumors of the urinary tract: a clinicopathologic study of 46 cases, including a malignant example inflammatory fibrosarcoma and a subset associated with high-grade urothelial carcinoma. *Am. J. Surg. Pathol*. **30,** 1502–1512.
12. Karam, J.A., Kabbani, W., and Sagalowsky, A.I. (2008) Pseudosarcomatous fibromyxoid tumor of the bladder. *Urol. Oncol*. **26,** 291–294.
13. Iczkowski, K.A., Shanks, J.H., Gadaleanu, V., et al. (2001) Inflammatory pseudotumor and sarcoma of urinary bladder: differential diagnosis and outcome in thirty-eight spindle cell neoplasms. *Mod. Pathol*. **14,** 1043–1051.
14. Proppe, K.H., Scully, R.E., and Rosai, J. (1984) Postoperative spindle cell nodules of genitourinary tract resembling sarcomas. A report of eight cases. *Am. J. Surg. Pathol.* **8,** 101–108.
15. Jensen, K.B., Langkilde, N.C., Lundbeck, F., et al. (2003) Pseudosarcomatous fibromyxoid tumor of the prostate. *Scand. J. Urol. Nephrol*. **37,** 85–87.
